# Risk of Sarcopenic Obesity Across Menopausal Transition Stages in Middle-Aged Korean Women

**DOI:** 10.3390/nu17203238

**Published:** 2025-10-15

**Authors:** Yoosun Cho, Yoonyoung Jang, Jae Ho Park, Yoosoo Chang, Seungho Ryu

**Affiliations:** 1 Center for Cohort Studies, Total Healthcare Center, Kangbuk Samsung Hospital, Sungkyunkwan University School of Medicine, Seoul 04514, Republic of Korea; misslonghorn46@gmail.com (Y.C.);; 2 Asan Medical Center, University of Ulsan College of Medicine, Seoul 05505, Republic of Korea; 3 Institute of Medical Research, Sungkyunkwan University School of Medicine, Suwon 16419, Republic of Korea; 4 Division of Population Health Research, Department of Precision Medicine, National Institute of Health, Cheongju 28161, Republic of Korea; 5 Department of Occupational and Environmental Medicine, Kangbuk Samsung Hospital, Sungkyunkwan University School of Medicine, Seoul 04514, Republic of Korea; 6 Department of Clinical Research Design and Evaluation, Samsung Advanced Institute for Health Sciences and Technology, Sungkyunkwan University, Seoul 06355, Republic of Korea

**Keywords:** sarcopenia, obesity, menopause, body composition, longitudinal studies, middle-aged

## Abstract

**Background/Objectives:** The risk of sarcopenic obesity across menopausal transition stages remains unclear. This study investigated the association between menopausal stage and the risk of sarcopenic obesity. **Methods:** This longitudinal study included 4766 Korean women aged 42–52 years (median follow-up duration, 9.1 years). Menopausal transition was classified by STRAW + 10 stages: premenopause, early transition, late transition, and postmenopause. Sarcopenic obesity was defined as ASM index < 5.7 kg/m^2^ combined with PBF ≥ 35%. ASM and PBF were measured by bioelectrical impedance analysis with an InBody 720 device. Obesity, defined by BMI or waist circumference, was used for the sensitivity analyses. Associations were analyzed using generalized estimating equations. **Results:** Participants had a mean age and BMI of 42.2 years (SD 3.0) and 22.1 kg/m^2^ (SD 2.9), respectively; 16.1% had high body fat, and 15.6% had low muscle mass. Increased age was associated with higher odds of sarcopenic obesity (OR 1.07, 95% CI: 1.03–1.10). Compared to pre-menopause, late transition (OR 1.49, 95% CI: 1.17–1.89) and post-menopause (OR 1.67, 95% CI: 1.26–2.23) were significantly associated with sarcopenic obesity, independent of confounders. Similar trends were observed using waist circumference-based definitions. No significant associations were identified using BMI-based definitions (BMI ≥ 23 kg/m^2^), although positive trends were observed. **Conclusions:** In middle-aged Korean women undergoing natural menopause, sarcopenic obesity increased across menopausal stages, becoming significant from the late transition. These unfavorable changes were more evident when using body composition measures, suggesting that BMI alone may underestimate such changes during menopausal transition.

## 1. Introduction

Women undergo significant changes in body composition during midlife, particularly during menopausal transition (MT) [1,2]. This period is marked by increased fat mass, including total and regional adipose tissue, and a reduction in lean mass [2]. Women experience an accelerated loss of muscle mass and strength at an earlier age than men during menopause [3]. A Finnish longitudinal study of middle-aged women reported that declining estradiol levels during MT can predict significant decreases in lean body mass, appendicular lean mass, and thigh muscle cross-sectional area, supporting the concept of accelerated muscle loss during this time [4]. Loss of muscle mass and function is known as sarcopenia, a progressive disorder associated with increased morbidity and mortality [5,6,7]. During aging and the MT, progressive muscle degeneration occurs [8], which can lead to sarcopenia. Sarcopenia is a progressive and generalized skeletal muscle disorder characterized by loss of muscle mass, strength, and function, increasing the risk of adverse outcomes, including falls, fractures, physical disability, and mortality [5,6,7,9].

When sarcopenia coexists with excess body fat, especially central adiposity, it is termed “sarcopenic obesity” [10]. Hormonal changes during menopause, such as reduced estrogen levels and increased follicle-stimulating hormone and androgen levels [11,12], play a major role in the development of sarcopenic obesity. Declining estrogen levels promote visceral fat accumulation and muscle loss, while triggering proinflammatory cytokines, which accelerate muscle protein breakdown and inhibit muscle synthesis [4,13]. These combined mechanisms synergistically increase the risk of sarcopenic obesity and increase susceptibility to morbidity and mortality [14,15,16,17]. Importantly, the risks associated with sarcopenic obesity exceed those associated with sarcopenia or obesity alone [18,19], highlighting the importance of elucidating the risk of sarcopenic obesity across MT, a potential window period for women’s physical health and function.

Although observational studies have reported the changes in body composition in peri- or post-menopause [2,20,21], often without specifically addressing the clinical and functional term sarcopenic obesity, evidence on sarcopenic obesity risk across distinct MT stages remains limited. Therefore, this study aimed to conduct a longitudinal analysis of sarcopenic obesity risk, defined by a validated Asian-specific cutoff for appendicular skeletal muscle mass (ASM), and multiple measures of obesity, including body mass index (BMI), waist circumference (WC), and percent body fat (PBF), across four MT stages (premenopause, early transition, late transition, and postmenopause) in middle-aged Korean women. We hypothesized that (1) the risk of sarcopenic obesity would increase progressively across MT stages, and (2) PBF or WC-based definitions would better represent these changes than BMI-based definitions.

## 2. Materials and Methods

This longitudinal study included women aged 42–52 years who underwent comprehensive health examinations at the Total Healthcare Center at Kangbuk Samsung Hospital between 2014 and 2018 [22,23], with follow-up extending until July 2024. At baseline, all participants were in the premenopausal or in the early transition stage and underwent regular annual or biennial health examinations [22,23]. All participants were informed in person regarding the purpose and design of the study and voluntarily provided written consent for the use of their retrospective and prospective health data. Eligibility at baseline was defined according to the following criteria: (1) no history of hysterectomy, oophorectomy, or hormone replacement therapy; (2) no diagnosis of underlying conditions, including kidney failure, malignancy, hypothyroidism, or hyperthyroidism; and (3) no history of amenorrhea lasting ≥60 days [22,23]. This study was approved by the Institutional Review Board of Kangbuk Samsung Hospital (KBSMC 2023-05-036-022).

### 2.1. Sample Description

**Figure 1:**
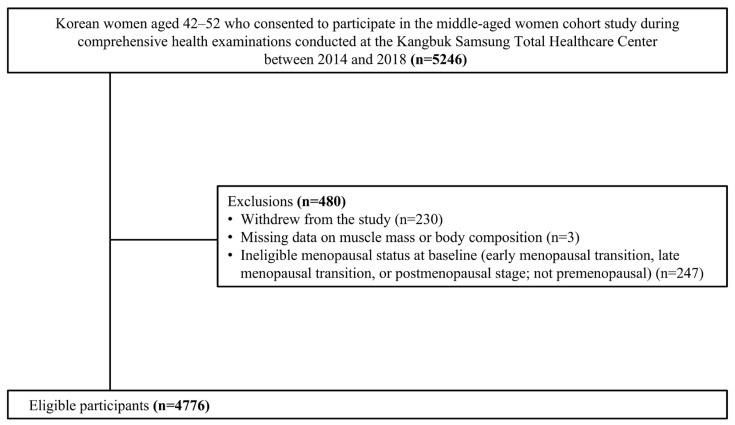
A flow chart for participant selection; A total of 4776 women were included in the analysis.

Figure 1 presents the participant flowchart. Of the 5246 participants initially enrolled, 4766 were included in the final analysis after excluding 480 (9.1%). The exclusion criteria were as follows: withdrawal of consent (*n* = 230; 4.4%), missing data on muscle mass (*n* = 3; 0.06%), and not being in the premenopausal stage at baseline (*n* = 247; 4.7%). The study participants were followed up for a median of 9.1 years (interquartile range [IQR], 6.3–10.8 years), with a median interval between follow-up visits of 1.0 year (IQR, 0.9–1.2 years).

### 2.2. Measurement

Demographic information, lifestyle factors, reproductive status, and medical history were collected using a standardized self-administered questionnaire. Body composition, including ASM and fat mass, was measured using BIA with an InBody 720 device (Biospace Co., Ltd., Seoul, Republic of Korea) [24]. The cutoff for PBF ≥ 35% was selected based on previous studies in Asian populations, reflecting evidence on metabolic risk and population-specific adiposity, and is consistent with widely adopted criteria in the recent literature [25,26]. Asian-specific thresholds for BMI categorized as normal (<23.0 kg/m^2^), overweight (23.0–24.9 kg/m^2^), and obese (≥25.0 kg/m^2^) and WC, classified as abdominal obesity (≥80 cm) or normal obesity (<80 cm), align with recommendations from the World Health Organization (WHO) and regional metabolic risk data [27,28]. The cutoff for the ASM index was derived from a large multi-cohort study in combination with the current consensus of the Asian Working Group for Sarcopenia, which defined low muscle mass as ASM index < 5.7 kg/m^2^ for women by BIA [10].

Age at menarche was categorized as <12, 12–13, 14–16, or ≥17 years. Smoking status was dichotomized as never smoker (<5 packs in a lifetime) or ever-smoker (≥5 packs) [29]. Alcohol consumption was classified as <10 g/day or ≥10 g/day [30]. Physical activity was categorized as inactive, minimally active, or active according to the Korean version of the short form of the International Physical Activity Questionnaire [31], with the active category meeting the criteria for Health-Enhancing Physical Activity [31]. Marital status was classified as unmarried, married/cohabiting, or divorced/separated/widowed. Parity was categorized as nulliparous or parous. Educational level was classified as high school or lower versus college or higher. Diabetes mellitus was defined as a fasting plasma glucose level ≥ 126 mg/dL, hemoglobin A1c level ≥ 6.5%, or a history of antidiabetic medication use [32]. Hypertension was defined as systolic blood pressure ≥ 140 mmHg, diastolic blood pressure ≥ 90 mmHg, or a history of blood pressure-lowering medication use [33]. Dyslipidemia was defined as low-density lipoprotein cholesterol level ≥ 160 mg/dL, triglyceride level ≥ 200 mg/dL, high-density lipoprotein cholesterol level < 40 mg/dL, or a history of lipid-lowering medication use [34].

### 2.3. Definition of Menopausal Stages

Menopausal stage and menstrual cycle information were prospectively assessed at each visit through a self-administered structured questionnaire. Menopausal stage was classified according to the Stages of Reproductive Aging Workshop (STRAW) + 10 criteria [16], based primarily on menstrual bleeding patterns. Four categories were defined: (1) pre-menopause: regular menstrual cycles without meeting any criteria for subsequent stages; (2) early transition: at least two menstrual cycles differing by ≥7 days in length; (3) late transition: amenorrhea lasting for ≥60 days; (4) post-menopause: no menstrual periods for ≥12 months.

To determine these stages, participants were asked standardized questions at each visit, including whether they had experienced a menstrual period in the past year; whether, during their last ten menstrual periods, they had at least two cycles differing by more than seven days; and whether they had experienced any menstrual cycles with an interval of more than 60 days in the past year. Based on the responses to these questions, menopausal stage was determined at each time point using the STRAW + 10 staging criteria.

Although follicle-stimulating hormone (FSH) and estradiol levels were not measured at every visit for all participants, a total of 969 measurements were available (212 in pre-menopause, 155 in early transition, 279 in late transition, and 323 in post-menopause). We summarized the distributions of these hormone levels across MT using the available measurements.

### 2.4. Definition of Sarcopenic Obesity

Sarcopenic obesity was defined using combinations of the ASM index and obesity indicators as follows [35,36,37]: (1) ASM index < 5.7 kg/m^2^ and PBF ≥ 35% and (2) ASM index < 5.7 kg/m^2^ and WC ≥ 80 cm. We used PBF ≥ 35% and WC ≥ 80 cm as obesity thresholds because these values have been associated with elevated cardiometabolic risk in Asian women, reflecting population-specific differences in body fat distribution [38,39,40]. Only 8 participants met the criterion of ASM index < 5.7 kg/m^2^ with BMI ≥ 25.0 kg/m^2^, and 15 participants met the criterion with WC ≥ 85 cm during follow-up, which were not available for the sensitivity analyses. Therefore, for sensitivity analyses, sarcopenic obesity was alternatively defined using the following criteria: (3) ASM index < 5.7 kg/m^2^ and BMI ≥ 23.0 kg/m^2^, and (4) ASM index < 5.7 kg/m^2^ and PBF ≥ 38%.

### 2.5. Statistical Analysis

Baseline characteristics are presented as means with standard deviations (SDs) for continuous variables and as frequencies and percentages for categorical variables. Generalized estimating equations (GEE) were applied to evaluate the association between MT and sarcopenic obesity, accounting for within-subject correlations due to repeated measurements. The models were fitted using an exchangeable working correlation structure and robust standard errors, with individual identifiers specified as clustering variables (xtgee command with a logit link function in STATA). Model selection was informed by the quasi-likelihood under the independence model criterion (QIC) (Table S6) [41]. We incorporated covariates that were causally relevant to sarcopenic obesity and either causally or non-causally associated with the MT, excluding those that could serve as intermediates in the causal pathway between MT and sarcopenic obesity [42].

In the main analysis (ASM index < 5.7 kg/m^2^ with PBF ≥ 35% or ASM index < 5.7 kg/m^2^ with WC ≥ 80 cm), model 1 was adjusted for time-varying age. Model 2 was adjusted for smoking status, physical activity, alcohol consumption, educational level, parity, marital status, and age at menarche. Model 3 further included hypertension, diabetes mellitus, and dyslipidemia, in addition to the covariates in model 2, to assess the potential mediation by comorbidities. Because this study primarily aimed to examine the association between MT and sarcopenic obesity, age and MT were treated as time-varying covariates, whereas all other covariates were considered time-fixed because these variables were not available at every visit.

Statistical power to detect an association between MT and sarcopenic obesity was evaluated by simulation-based estimation using the fitted GEE logistic regression model (binomial family, exchangeable correlation structure) implemented in the geepack package in R (version 4.4.2), with a Wald test from the aod package. The outcome variable was re-generated 2000 times based on the fitted model, and the proportion of simulations rejecting the null hypothesis (H_0_: no MT effect) at α = 0.05 was taken as the statistical power [43].

We conducted a series of sensitivity analyses to assess the robustness of our findings: (1) treating all covariates except age at menarche as time-varying variables, with missing data addressed using the mice package in R; (2) performing a lagged analysis in which MT stages and covariates were measured at time t and sarcopenic obesity at time t + 1; (3) redefining sarcopenic obesity using alternative cut-offs for BMI (≥23.0 kg/m^2^) or PBF (≥38%); (4) restricting the analysis to women who were cardiometabolically healthy at baseline, with missing data addressed using multiple imputation (*n* = 4224); (5) modeling changes in PBF and WC as continuous outcomes in relation to time from the final menstrual period among participants who had reached menopause (*n* = 1752), using cubic restricted spline models with the splines package in R; and (6) redefining sarcopenic obesity according to the Asian female criteria, using weight-adjusted appendicular skeletal muscle mass (ASM; 26.4% or 29.6%) combined with obesity defined by either PBF ≥ 35% or WC ≥ 80 cm as the outcome [44].

Regarding the handling of missing data, in the primary analyses, missing values in categorical variables were addressed by creating separate “unknown” categories, and Stata’s factor-variable syntax (i. prefix) automatically generated the corresponding indicator variables during estimation. In sensitivity analysis 2, all covariates except age at menarche were treated as time-varying variables. Missing data proportions were relatively low for most covariates (≤9.9%) throughout the follow-up period. Little’s test rejected the MCAR assumption, suggesting that missingness was plausibly Missing at Random (MAR) [45]. Because the overall proportion of missingness was below the commonly accepted threshold of approximately 20% for reliable multiple imputation [45], we applied multiple imputation using the mice package in R [46]. Five imputed datasets (m = 5) were generated, generalized estimating equation (GEE) logistic regression models were fitted to each dataset, and estimates were pooled using Rubin’s rules [46]. Dietary questionnaire data were excluded due to >60% missingness.

Statistical analyses were performed using STATA version 18 (StataCorp LLC, College Station, TX, USA) and R (version 4.4.2). A two-sided *p*-value of <0.05 was considered statistically significant.

## 3. Results

Table 1 presents the baseline characteristics of the study population. The mean age and BMI were 42.2 (SD, 3.0) and 22.1 kg/m^2^ (SD, 2.9), respectively.

To visualize the hormonal changes across MT (the main exposure of this study), we examined the 969 available hormone measurements and observed that FSH levels increased while estradiol levels decreased across the transition, with the most pronounced changes occurring between the early and late transition stages (Figure S1a,b). In addition, we showed the predicted probability of sarcopenic obesity by MT at fixed ages (45 and 50 years). The predicted probability was higher at age 50 than at age 45, but the overall pattern of change across MT was similar in both age groups (Figure S2).

**Figure 2:**
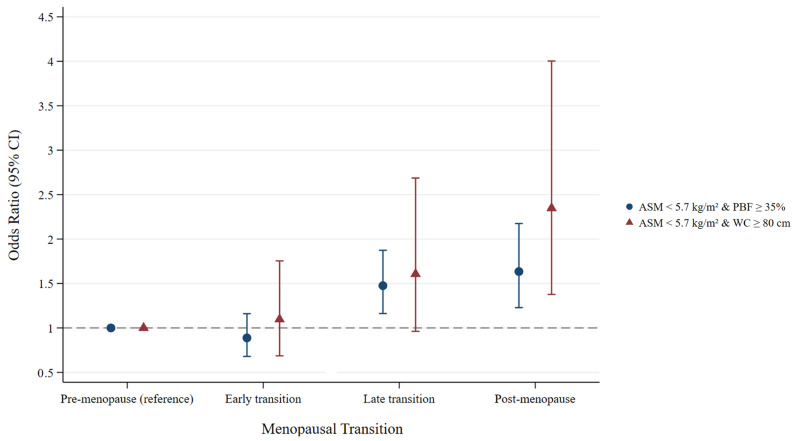
Risk of sarcopenic obesity defined by low muscle mass and excessive adiposity across menopausal transition stages. The model was adjusted for time-varying age, with all other covariates treated as time-fixed variables (smoking status, regular physical activity, alcohol consumption, education level, parity, marital status, and age at menarche). Sarcopenic obesity was defined as low muscle mass (ASM < 5.7 kg/m^2^) combined with excessive adiposity (PBF ≥ 35% or WC ≥ 80 cm). Abbreviations: ASM, appendicular skeletal muscle; PBF, percent body fat; WC, waist circumference.

Table 2 presents the odds ratios (ORs) for the association between MT and sarcopenic obesity, defined as a combination of ASM < 5.7 kg/m^2^ and PBF ≥ 35%. Increasing age was associated with higher odds of sarcopenic obesity, with an OR of 1.07 (95% confidence interval [CI]: 1.04–1.10). Compared with the premenopausal stage, both the late transition and postmenopausal stages were significantly associated with increased odds of sarcopenic obesity. In age-adjusted model, the ORs were 1.47 (95% CI: 1.16–1.87) for the late transition stage and 1.63 (95% CI: 1.22–2.16) for the postmenopausal stage. These associations remained consistent after further adjustment for sociodemographic and lifestyle covariates (Table 2, Figure 2). Using the definition of sarcopenic obesity as ASM < 5.7 kg/m^2^ combined with WC ≥ 80 cm, the postmenopausal stage remained significantly associated with sarcopenic obesity, with ORs of 2.27 (95% CI: 1.33–3.88) in Model 1 and 2.35 (95% CI: 1.38–4.00) in Model 2 (Table 3, Figure 2). Time-varying age was not significantly associated with sarcopenic obesity in either model.

The estimated statistical power was 0.975 for sarcopenic obesity defined as ASM < 5.7 kg/m^2^ with PBF ≥ 35% and 0.876 for sarcopenic obesity defined as ASM < 5.7 kg/m^2^ with WC ≥ 80 cm, indicating adequate power for both outcome definitions.

When sarcopenic obesity was defined using ASM and BMI ≥ 23.0 kg/m^2^ (Table 4), no significant associations were observed between MT and sarcopenic obesity, although positive trends were noted in the late transition and postmenopausal stages. In contrast, defining sarcopenic obesity using ASM and PBF ≥ 38% yielded associations consistent with those observed in the primary analysis (Table 4).

We conducted six sensitivity analyses. When the models were further adjusted for hypertension, diabetes mellitus, and dyslipidemia beyond the covariates included in model 2 in sensitivity analysis 1, the association between MT and sarcopenic obesity remained similar across all definitions used, although the ORs were slightly attenuated (defined by ASM < 5.7 kg/m^2^ with either PBF ≥ 35% or WC ≥ 80 cm) (Table S1).

According to MI-based sensitivity analysis 2, the results were consistent with those of the primary analyses (Table S2). In sensitivity analysis 3, which was based on the lagged analysis, MT stage was associated with an increased risk of sarcopenic obesity. When sarcopenic obesity was defined as ASM < 5.7 kg/m^2^ combined with PBF ≥ 35%, both the late transition and postmenopause stages were significantly associated with a higher risk compared with the premenopause stage. When defined as ASM < 5.7 kg/m^2^ combined with WC ≥ 80 cm, the postmenopause stage showed a significant association. The estimates were consistent with those observed in the primary analysis (Table S3).

According to sensitivity analysis 4, among 4224 participants who were cardiometabolically healthy at baseline, after excluding 542 participants (11.4%) with a history of hypertension, diabetes, or dyslipidemia, while addressing missing data using multiple imputation, the odds ratios for late transition and postmenopause in relation to sarcopenic obesity defined as ASM < 5.7 kg/m^2^ with PBF ≥ 35% were slightly attenuated but remained statistically significant and consistent with the primary results (Table S4).

Based on cubic restricted spline models in sensitivity analysis 5, PBF showed a consistently increasing pattern from 11 years before to 9 years after the FMP (Figure S3), whereas WC increased up to approximately 3 years before the FMP, plateaued until about 3 years after the FMP, and then resumed a relatively steeper upward trajectory thereafter (Figure S4).

In sensitivity analysis 6, when sarcopenic obesity was defined using weight-adjusted ASM, its association with MT was not statistically significant (Table S5).

## 4. Discussion

In this longitudinal study of women undergoing MT, the risk of sarcopenic obesity, defined by elevated PBF or large WC, was associated with an advanced menopausal stage, whereas no significant association was observed when BMI was used as the adiposity criterion. Sarcopenic obesity risk increased significantly from the late transition stage and was most evident when defined by PBF (>35%). Thus, BMI alone may be less accurate in tracking changes in body composition during this period. These associations remained significant after adjusting for age and other cardiometabolic comorbidities. Women in the MT group had an increased risk of sarcopenic obesity prior to reaching menopause.

Previous cohort studies have reported varying results regarding MT-related changes in body composition. For example, Sowers et al., using BIA in a sample of 130 women at the Michigan Study of Women’s Health Across the Nation (SWAN) site, observed no effect of final menstrual period (FMP) timing on fat or lean mass; instead, they observed a linear increase in fat mass and a modest linear decrease in lean mass over time [20]. Similarly, the MONET study of 48 women indicated that neither weight nor BMI was influenced by FMP timing; the percent fat mass was higher in the post-FMP years, although no change was observed during the transition before FMP [21]. Davies et al. also noted a linear increase in weight over time with no specific effect of FMP timing [47]. However, these studies were limited by small sample sizes and a lack of detailed categorization of the MT stages, which may have contributed to the inconsistent findings.

Consistent with our findings, the longitudinal SWAN used dual-energy X-ray absorptiometry (DXA) data to assess body composition changes across the MT in relation to time since the FMP [2]. This study indicated that women experience a significant increase in fat mass and a decrease in lean mass as they progress through MT, with the most pronounced changes occurring during late transition and postmenopause, independent of aging effects, although no measurable change in body weight trajectory was observed during MT [2]. In contrast to previous studies that primarily assessed the associations between components of body composition (fat mass, lean mass, and body weight) and MT, our study used a validated Asian-specific cutoff for appendicular skeletal muscle mass and incorporated multiple measures of obesity (BMI, WC, and PBF) into the clinical construct of sarcopenic obesity. Additionally, we assessed the risk across detailed menopausal stages, distinguishing between early and late transitions, which provides a more refined understanding of sarcopenic obesity risk throughout the MT.

In our study, sarcopenic obesity, as defined by BMI, was not associated with MT. One potential explanation is the low prevalence of BMI-based obesity, especially the very small number of cases combining obesity with low muscle mass (only eight women met this criterion), which limited further analysis. However, even among women with normal BMI, obesity based on body composition combined with low muscle mass increased significantly across the MT stages, highlighting the limitations of BMI in detecting adiposity-related health risks during this critical period. Supporting this, the Framingham Heart Study reported that visceral adiposity, rather than BMI, was more strongly associated with incident diabetes, hypertension, low high-density lipoprotein levels, and cardiovascular events, including cardiovascular disease-related mortality in women [48]. BMI encompasses both fat and muscle, which does not sufficiently account for changes in body composition during MT. For example, data from the SWAN cohort demonstrated that MT was accompanied by accelerated gains in fat mass and simultaneous losses in lean mass; their joint rates of change resulted in no detectable acceleration in weight or BMI at the onset of MT [2]. BMI remained unchanged because concurrent fat gain and lean mass loss counterbalanced each other, masking clinically relevant changes in body composition. This observation suggests BMI alone may underestimate sarcopenic obesity risk during the MT. Furthermore, sarcopenic obesity, defined using visceral fat attenuation, was associated with poorer survival rates regardless of BMI [49]. These findings highlight the importance of sex-specific body fat distribution and hormonal changes that are not captured by BMI alone. Therefore, our results support the growing consensus that although BMI remains a standard cardiometabolic risk indicator, it lacks sensitivity to clinically relevant shifts in adiposity and fat distribution [50], particularly in midlife women undergoing MT.

Several mechanisms link menopause to the development of sarcopenic obesity. A decline in estradiol levels, which is essential for maintaining muscle mass and regulating fat distribution, leads to increased visceral fat and reduced muscle strength. Skeletal muscle contains specific estrogen receptors that stimulate satellite cell proliferation and promote muscle regeneration [51]; thus, declining estrogen levels during menopause directly impair muscle maintenance and recovery. Estrogen deficiency also drives the redistribution of adipose tissue from the peripheral subcutaneous depots, especially the gluteofemoral region, to the central visceral compartments [52]. Obesity activates immune cells, such as macrophages, mast cells, and T lymphocytes, triggering a chronic inflammatory response. This inflammatory response increases the secretion of proinflammatory cytokines, including tumor necrosis factor (TNF), interleukin-6 (IL-6), and leptin, while levels of insulin-like growth factor-1 (IGF-1) typically decrease with age and obesity. Together, these changes contribute to insulin resistance, muscle catabolism, and fat accumulation [18,53]. In addition, elevated leptin and reduced adiponectin levels worsen inflammation, hinder muscle growth, and decrease fat oxidation, thereby promoting sarcopenic obesity [18]. Collectively, these interconnected pathways create a vicious cycle that exacerbates the adverse effects of menopause on body composition.

## 5. Limitations of the Study

This study had some limitations. First, our operational definition of sarcopenic obesity was based on multifrequency, segmental bioelectrical impedance analysis (BIA; InBody 720, Biospace Co., Ltd., Seoul, Republic of Korea). Although BIA is practical and widely used, its estimates can be influenced by hydration status and by device- and population-specific prediction equations, with performance varying across devices [54]. In comparative studies—including evaluations of InBody analyzers—agreement with DXA has been acceptable at the group level but shows systematic bias and relatively wide individual limits of agreement, which can lead to misclassification in some individuals [55]. Accordingly, our findings should be interpreted with appropriate caution; future studies may benefit from incorporating study-specific calibration against DXA or CT or reporting device-specific error metrics in line with current consensus recommendations [9]. However, in our study, we utilized previously collected data in which body composition had already been measured using BIA—a non-invasive, quick, radiation-free, cost-effective, and portable method that allows efficient repeated assessments. Moreover, BIA is more practical and feasible for routine use in clinical settings, making it suitable for large-scale screening and longitudinal monitoring. It is a validated and widely used technique in population-based studies to estimate body composition [56,57,58]. Second, muscle strength data (e.g., grip strength) or physical performance measures (e.g., gait speed, chair rise tests) were not available. The absence of these functional assessments limits the clinical interpretation of sarcopenic obesity, given that current consensus definitions emphasize both structural and functional components of sarcopenia. Further studies are needed to strengthen our findings by incorporating muscle strength or physical performance criteria into the definition of sarcopenic obesity. Including tests such as handgrip strength, gait speed, or chair-stand tests alongside body composition measurements will enhance diagnostic accuracy and improve risk stratification for adverse health outcomes. Third, STRAW + 10 criteria based on self-reported menstrual history and clinical data. While this approach is standard, some misclassification is possible due to recall bias. Exclusion criteria and the large cohort size help reduce major errors, but potential classification inaccuracies remain a recognized limitation that should be considered when interpreting the results. Fourth, although the application of multiple imputation for missing data yielded results consistent with those of the primary analyses, dietary information, including total calorie intake, was unavailable since its proportion of missingness far exceeded the commonly acceptable threshold for multiple imputation (approximately 20%) [45]. As dietary factors may confound the association between MT and the risk of sarcopenia, some residual bias in the point estimates may still remain due to potential unmeasured confounding. Fifth, in the primary analysis, we used height-adjusted ASM because previous studies have reported that this index is more strongly correlated with muscle function than weight- or BMI-adjusted indices [59]. However, further longitudinal research in menopausal women is warranted to clarify how the choice between height- and weight-adjusted indices influences observed changes in body composition and their clinical implications across the MT. Finally, our study population consisted of healthy, middle-aged Korean adults with good access to healthcare, which may limit generalizability. Given that participants were predominantly highly educated, our sample may underrepresent individuals from lower socioeconomic backgrounds or varied ethnicities. This selection characteristic is an inherent limitation, reflecting a healthy volunteer effect where participants often have better health awareness and behaviors than the general population. Therefore, caution is needed when applying these findings to populations with different socioeconomic or ethnic backgrounds. Future studies involving broader population samples would help evaluate the wider applicability of these results. Nevertheless, the strengths of our study are the use of a clinically relevant and commonly available practical definition of sarcopenic obesity that enhances the applicability of our findings to real-world settings; its longitudinal design with a large sample size; detailed menopausal staging; adjustment for time-varying age and MT; and the use of statistical methods appropriate for repeated measurements.

Specifically, we propose combining ASM index measurement with adiposity assessment using PBF (≥35%) or WC (≥80 cm). Women with low ASM in combination with elevated PBF or WC should be considered at risk of sarcopenic obesity and counseled for targeted interventions, including resistance exercise, dietary optimization, and regular monitoring. Supporting this, a cohort study demonstrated positive associations between physical activity and lean mass, with higher activity levels significantly linked to greater appendicular lean mass across premenopausal, perimenopausal, and postmenopausal stages [4,60]. Furthermore, combined exercise and nutrition interventions have proven effective in improving sarcopenia-related outcomes in older adults [61]. Incorporating screening into routine annual health checkups for women undergoing MT would facilitate early detection and timely management.

## 6. Conclusions

The risk of sarcopenic obesity based on WC and PBF, but not BMI, increased significantly from the late MT stage, and this association was independent of chronological aging. Early identification of sarcopenic obesity in middle-aged women undergoing MT may facilitate timely intervention to prevent adverse changes in body composition and preserve musculoskeletal health, thereby reducing the risk of long-term complications. Importantly, reliance on BMI alone may overlook women at high risk during MT, highlighting the need to incorporate PBF or WC in clinical screening strategies.

## Figures and Tables

**Table 1:** Baseline characteristics of the study population (*n* = 4766).

Variable	Frequency (%)
Age at baseline ^†^	42.2 ± 3.0
Age at menarche	
<12 years old	169 (3.6)
12–13 years old	1665 (34.9)
14–16 years old	2666 (55.9)
≥17 years old	184 (3.9)
Unknown	82 (1.7)
Smoking	
Non-smoker	4072 (85.4)
Ever-smoker	533 (11.2)
Unknown	161 (3.4)
Alcohol consumption	
<10 g/day	3918 (82.2)
≥10 g/day	403 (8.5)
Unknown	445 (9.3)
Parity	
Nulliparous	332 (7.0)
Parous	4282 (89.8)
Unknown	152 (3.2)
Marital status	
Unmarried	207 (4.3)
Married/cohabitating	4378 (91.9)
Divorced/separated/widowed	78 (1.6)
Unknown	103 (2.2)
Education	
≤High school	960 (20.2)
≥College	3629 (76.1)
Unknown	177 (3.7)
Physical activity ^‡^	
Active ^§^	543 (11.4)
Minimally active	1795 (37.7)
Inactive	2343 (49.1)
Unknown	85 (1.8)
Hypertension ^¶^	144 (3.0)
Diabetes mellitus ^††^	79 (1.7)
Hypercholesterolemia ^‡‡^	389 (8.2)
Low muscle mass ^§§^	742 (15.6)
Obesity	
PBF ≥ 35%	767 (16.1)
WC ≥ 80 cm	1348 (28.3)
BMI ≥ 23 kg/m^2^	1487 (31.2)
Sarcopenic obesity	
ASM < 5.7 kg/m^2^ + PBF ≥ 35%	65 (1.4)
ASM < 5.7 kg/m^2^ + WC ≥ 80 cm	24 (0.5)
ASM < 5.7 kg/m^2^ + BMI ≥ 23 kg/m^2^	13 (0.3)

^†^ Age at baseline is presented as means and standard deviations. ^‡^ The Korean version of the short form of the International Physical Activity Questionnaire was used to assess physical activity. ^§^ High physical activity levels indicate health-enhancing physical activity. ^††^ Diabetes mellitus was defined as a fasting plasma glucose level of ≥126 mg/dL, a hemoglobin A1c (HbA1c) level of ≥6.5%, or a history of antidiabetic medication use. **^¶^** Hypertension was defined as a systolic blood pressure ≥ 140 mmHg, diastolic blood pressure ≥ 90 mmHg, or a history of blood pressure-lowering medication use. ^‡‡^ Dyslipidemia was defined as a low-density lipoprotein cholesterol level ≥ 160 mg/dL, triglyceride level ≥ 200 mg/dL, high-density lipoprotein cholesterol level < 40 mg/dL, or a history of lipid-lowering medication use. ^§§^ Low muscle mass was defined as appendicular skeletal muscle mass < 5.7 kg/m^2^. Abbreviations: PBF, percent body fat; BMI, body mass index; WC, waist circumference; ASM, appendicular skeletal muscle mass.

**Table 2:** Risk of sarcopenic obesity defined by percent body fat across menopausal transition stages.

ASM < 5.7 kg/m^2^ and PBF ≥ 35%	Model 1 ^†^		Model 2 ^‡^	
	Adjusted OR (95% CI)	*p*-Value	Adjusted OR (95% CI)	*p*-Value
Time-varying age (year)	1.07 (1.04–1.10)	<0.001	1.07 (1.04–1.10)	<0.001
Menopausal transition				
Premenopause	Ref (1)	–	Ref (1)	–
Early transition	0.89 (0.68–1.16)	0.375	0.89 (0.68–1.16)	0.384
Late transition	1.47 (1.16–1.87)	<0.001	1.48 (1.16–1.87)	<0.001
Postmenopause	1.63 (1.22–2.16)	<0.001	1.63 (1.23–2.17)	<0.001

^†^ Model 1 included only time-varying age. ^‡^ Model 2 included time-varying age, with all other covariates treated as time-fixed variables (smoking status, regular physical activity, alcohol consumption, education level, parity, marital status, and age at menarche). Abbreviations: ASM, appendicular skeletal muscle; PBF, percent body fat; OR, odds ratio; CI, confidence interval.

**Table 3:** Risk of sarcopenic obesity defined by abdominal obesity across menopausal transition stages.

ASM < 5.7 kg/m^2^ and WC ≥ 80 cm	Model 1 ^†^		Model 2 ^‡^	
	Adjusted OR (95% CI)	*p*-Value	Adjusted OR (95% CI)	*p*-Value
Time-varying age (year)	1.00 (0.95–1.05)	0.944	1.00 (0.95–1.05)	0.944
Menopausal transition				
Premenopause	Ref (1)	–	Ref (1)	–
Early transition	1.09 (0.69–1.72)	0.717	1.10 (0.69–1.75)	0.699
Late transition	1.57 (0.94–2.61)	0.082	1.61 (0.96–2.69)	0.070
Postmenopause	2.27 (1.33–3.88)	0.003	2.35 (1.38–4.00)	0.002

^†^ Model 1 included only time-varying age; ^‡^ Model 2 included time-varying age, with all other covariates treated as time-fixed variables (smoking status, regular physical activity, alcohol consumption, education level, parity, marital status, and age at menarche). Abbreviation: ASM, appendicular skeletal muscle; WC, waist circumference; OR, odds ratio; CI, confidence interval.

**Table 4:** Risk of sarcopenic obesity across menopausal transition stages using alternative definitions.

ASM < 5.7 kg/m^2^ and BMI ≥ 23 kg/m^2^	Model 1 ^†^		Model 2 ^‡^	
	Adjusted OR (95% CI)	*p*-Value	Adjusted OR (95% CI)	*p*-Value
Time-varying age (year)	1.06 (1.00–1.12)	0.038	1.06 (1.01–1.13)	0.025
Menopausal transition				
Premenopause	Ref (1)	–	Ref (1)	–
Early transition	0.96 (0.61–1.50)	0.861	0.94 (0.59–1.48)	0.781
Late transition	1.45 (0.86–2.44)	0.165	1.45 (0.85–2.47)	0.167
Postmenopause	1.40 (0.82–2.40)	0.214	1.44 (0.84–2.47)	0.180
**ASM < 5.7 kg/m^2^ and PBF ≥ 38%**	**Model 1 ^†^**		**Model 2 ^‡^**	
	**Adjusted OR (95% CI)**	***p*-Value**	**Adjusted OR (95% CI)**	***p*-Value**
Time-varying age (year)	1.06 (1.01–1.12)	0.014	1.07 (1.02–1.12)	0.008
Menopausal transition				
Premenopause	Ref (1)	–	Ref (1)	-
Early transition	0.99 (0.64–1.52)	0.946	0.97 (0.63–1.49)	0.898
Late transition	1.98 (1.17–3.36)	0.011	1.97 (1.16–3.33)	0.012
Postmenopause	2.15 (1.25–3.71)	0.006	2.16 (1.26–3.70)	0.005

^†^ Model 1 included only time-varying age; ^‡^ Model 2 included time-varying age, with all other covariates treated as time-fixed variables (smoking status, regular physical activity, alcohol consumption, education level, parity, marital status, and age at menarche). Abbreviations: ASM, appendicular skeletal muscle; BMI, body mass index; OR, odds ratio; CI, confidence interval.

## Data Availability

The data presented in this study are available on request from the corresponding author due to Institutional Review Board restrictions (the data were not collected in a way that could be widely distributed).

## Supplementary Materials

The following supporting information can be downloaded at https://www.mdpi.com/article/10.3390/nu17203238/s1. Table S1. Sensitivity analysis of sarcopenic obesity risk across menopausal transition stages adjusting for age and other confounders, with further adjustment for comorbidities. Table S2. Associations of menopausal transition with sarcopenic obesity from models with time-varying covariates and multiple imputation for missing data. Table S3. Associations of menopausal transition with risk of sarcopenic obesity at subsequent visits. Table S4. Associations of menopausal transition with sarcopenic obesity from models with time-varying covariates and multiple imputation for missing data, after excluding participants with cardiometabolic disease at baseline (*n* = 4224). Table S5. Risk of sarcopenic obesity defined by weight-adjusted ASM and obesity criteria across menopausal transition stages. Table S6. Goodness-of-fit diagnostics for GEE models with different working correlation structures. Figure S1. Distributions of FSH and Estradiol across menopausal stages. Figure S2. Predicted Probability of Sarcopenic Obesity by Menopausal Transition Stages at Fixed Ages (45 and 50 Years). Figure S3. Predicted percentage body fat by years relative to the final menstrual period, estimated using cubic restricted spline models. Figure S4. Predicted waist circumference relative to the final menstrual period, estimated using cubic restricted spline models.

## Abbreviations

ASM	Appendicular skeletal muscle mass
BIA	Bioelectrical impedance analysis
BMI	Body mass index
CI	Confidence interval
DXA	Dual-energy X-ray absorptiometry
FMP	Final menstrual period
FSH	Follicle-stimulating hormone
GEE	Generalized estimating equation
IQR	Interquartile range
MI	Multiple imputation
MT	Menopausal transition
OR	Odds ratio
PBF	Percent body fat
SD	Standard deviation
STRAW + 10	Stages of Reproductive Aging Workshop + 10 criteria
WC	Waist circumference
