# Supplementary material for: Risk of Sarcopenic Obesity Across Menopausal Transition Stages in Middle-Aged Korean Women
Source: Nutrients. 2025 Oct 15;17(20):3238. doi: 10.3390/nu17203238 (PMC12567333; doi:10.3390/nu17203238)

Table S1. Sensitivity analysis of sarcopenic obesity risk across menopausal transition stages adjusting for age and other confounders, with further adjustment for comorbidities.

**ASM < 5.7 kg/m²<br>and PBF ≥ 35%**	**Model 3 †**	
	**Adjusted OR (95% CI)**	***p*-Value**
Time-varying age (year)	1.07 (1.04-1.10)	< 0.001
Menopausal transition		
Premenopause	Ref (1)	–
Early transition	0.88 (0.68-1.15)	0.360
Late transition	1.47 (1.16-1.86)	0.002
Postmenopause	1.62 (1.22-2.16)	< 0.001
**ASM < 5.7 kg/m²<br>and WC ≥ 80 cm**	**Model 3**	
	**Adjusted OR (95% CI)**	***p*-Value**
Time-varying age (year)	1.00 (0.95-1.05)	0.983
Menopausal transition		
Premenopause	Ref (1)	–
Early transition	1.09 (0.68-1.74)	0.724
Late transition	1.59 (0.95-2.66)	0.076
Postmenopause	2.32 (1.36-3.95)	0.002
**ASM < 5.7 kg/m²<br>and BMI ≥ 23 kg/m²**	**Model 3**	
	**Adjusted OR (95% CI)**	***p*-Value**
Time-varying age (year)	1.06 (1.01-1.12)	0.024
Menopausal transition		
Premenopause	Ref (1)	–
Early transition	0.93 (0.59-1.47)	0.770
Late transition	1.45 (0.85-2.46)	0.170
Postmenopause	1.44 (0.84-2.47)	0.182

† Model 3 included time-varying age with all other covariates treated as time-fixed variables (smoking status, regular physical activity, alcohol consumption, education level, parity, marital status, age at menarche, hypertension, diabetes mellitus, and hypercholesterolemia).

Abbreviations: ASM, appendicular skeletal muscle; PBF, percent body fat; BMI, body mass index; WC, waist circumference; OR, odds ratio; CI, confidence interval.

Table S2. Associations of menopausal transition with sarcopenic obesity from models with time-varying covariates and multiple imputation for missing data.

	**ASM < 5.7 kg/m^2 & PBF ≥ 35%**		**ASM < 5.7 kg/m^2 & WC ≥ 80 cm**	
	**Adjusted OR (95% CI)**	***p*-Value**	**Adjusted OR (95% CI)**	***p*-Value**
Time-varying Age (Year)	1.00 (0.99-1.00)	0.065	1.00 (0.99-1.00)	0.492
Menopausal Transition				
Premenopause	Ref (1)	-	Ref (1)	-
Early transition	1.12 (0.88-1.44)	0.339	1.13 (0.70-1.83)	0.606
Late transition	2.07 (1.70-2.52)	< 0.001	1.65 (1.03-2.65)	0.037
Postmenopause	2.73 (2.23-3.34)	< 0.001	2.46 (1.49-4.05)	< 0.001

This model included time-varying covariates (age, smoking status, regular physical activity, alcohol consumption, education level, parity, and marital status), except for age at menarche, which was treated as time-fixed.
Abbreviation: ASM Appendicular Skeletal Muscle; PBF Percent Body Fat; OR Odds Ratio; CI Confidence Interval

Table S3. Associations of menopausal transition with risk of sarcopenic obesity at subsequent visits.

	ASM < 5.7 kg/m² & PBF ≥ 35%		ASM < 5.7 kg/m² & WC ≥ 80 cm	
	**Adjusted OR (95% CI)**	***p*-Value**	**Adjusted OR (95% CI)**	***p*-Value**
Time-varying Age (Year)	1.07 (1.04-1.11)	< 0.001	1.03 (0.97-1.09)	0.367
Menopausal Transition				
Premenopause	Ref (1)	-	Ref (1)	-
Early transition	1.06 (0.84-1.35)	0.623	1.28 (0.81-2.04)	0.291
Late transition	1.58 (1.24-2.01)	< 0.001	1.50 (0.87-2.57)	0.143
Postmenopause	1.58 (1.17-2.12)	0.003	2.19 (1.26-3.81)	0.006

This model included time-varying age, with all other covariates treated as time-fixed variables (smoking status, regular physical activity, alcohol consumption, education level, parity, marital status, and age at menarche).
Abbreviation: ASM Appendicular Skeletal Muscle; PBF Percent Body Fat; OR Odds Ratio; CI Confidence Interval

Table S4. Associations of menopausal transition with sarcopenic obesity from models with time-varying covariates and multiple imputation for missing data, after excluding participants with cardiometabolic disease at baseline (n=4224).

	**ASM < 5.7 kg/m² & PBF ≥ 35%**		**ASM < 5.7 kg/m² & WC ≥ 80 cm**	
	**Adjusted OR (95% CI)**	***p*-Value**	**Adjusted OR (95% CI)**	***p*-Value**
Time-varying Age (Year)	1.08 (1.04- 1.11)	< 0.001	0.99 (0.94-1.05)	0.787
Menopausal Transition				
Premenopause	Ref (1)	-	Ref (1)	-
Early transition	0.90 (0.69-1.19)	0.471	1.24 (0.78- 1.95)	0.358
Late transition	1.46 (1.14-1.88)	0.003	1.79 (1.07- 2.99)	0.025
Postmenopause	1.61 (1.19-2.18)	0.002	2.56 (1.52- 4.30)	< 0.001

Cardiometabolic diseases were defined as hypertension, diabetes, and dyslipidemia.
This model included time-varying covariates (age, smoking status, regular physical activity, alcohol consumption, education level, parity, and marital status), except for age at menarche, which was treated as time-fixed.
Abbreviation: ASM Appendicular Skeletal Muscle; PBF Percent Body Fat; OR Odds Ratio; CI Confidence Interval

Table S5. Risk of sarcopenic obesity defined by weight-adjusted ASM and obesity criteria across menopausal transition stages.

	**ASM/Wt < 26.4% & PBF ≥ 35%**		**ASM/Wt < 26.4% & WC ≥ 80 cm**	
	**Adjusted OR (95% CI)**	***p*-Value**	**Adjusted OR (95% CI)**	***p*-Value**
Time-varying Age (Year)	1.07 (1.06-1.08)	< 0.001	1.06 (1.05-1.07)	< 0.001
Menopausal Transition				
Premenopause	Ref (1)	-	Ref (1)	-
Early transition	1.02 (0.95-1.09)	0.664	1.00 (0.92-1.07)	0.901
Late transition	1.07 (0.98-1.16)	0.127	1.00 (0.92-1.10)	0.926
Postmenopause	1.08 (0.98-1.20)	0.126	1.04 (0.93-1.16)	0.479
	**ASM/Wt < 29.6% & PBF ≥ 35%**		**ASM/Wt < 29.6% & WC ≥ 80 cm**	
	**Adjusted OR (95% CI)**	***p*-Value**	**Adjusted OR (95% CI)**	***p*-Value**
Time-varying Age (Year)	1.07 (1.06-1.08)	< 0.001	1.03 (1.02-1.04)	< 0.001
Menopausal Transition				
Premenopause	Ref (1)	-	Ref (1)	-
Early transition	1.01 (0.94-1.08)	0.847	0.99 (0.94-1.05)	0.779
Late transition	1.08 (1.00-1.16)	0.058	1.02 (0.96-1.09)	0.487
Postmenopause	1.09 (0.99-1.20)	0.082	1.06 (0.97-1.15)	0.210

This model included time-varying age, with all other covariates treated as time-fixed variables (smoking status, regular physical activity, alcohol consumption, education level, parity, marital status, and age at menarche).

Abbreviation: ASM, appendicular skeletal muscle; Wt, weight; WC, waist circumference; OR, odds ratio; CI, confidence interval.

Table S6. Goodness-of-fit diagnostics for GEE models with different working correlation structures.

Quasi-likelihood under the Independence model Criterion (QIC)	Model 1 †	Model 2 ‡
**ASM < 5.7 kg/m^2 and PBF ≥ 35%**	Exchangeable: 7462<br>Independence: 7470	Exchangeable: 7420<br>Independence: 7442
**ASM < 5.7 kg/m^2 and WC ≥ 80 cm**	Exchangeable: 2276<br>Independence: 2279	Exchangeable: 2267<br>Independence: 2270

† Model 1 included only time-varying age

‡ Model 2 included time-varying age, with all other covariates treated as time-fixed variables (smoking status, regular physical activity, alcohol consumption, education level, parity, marital status, and age at menarche).

The exchangeable working correlation structure yielded a lower QIC than the independence structure, indicating a better model fit.

Abbreviation: ASM, appendicular skeletal muscle; WC, waist circumference.

Figure S1. Distributions of FSH and Estradiol across menopausal stages.

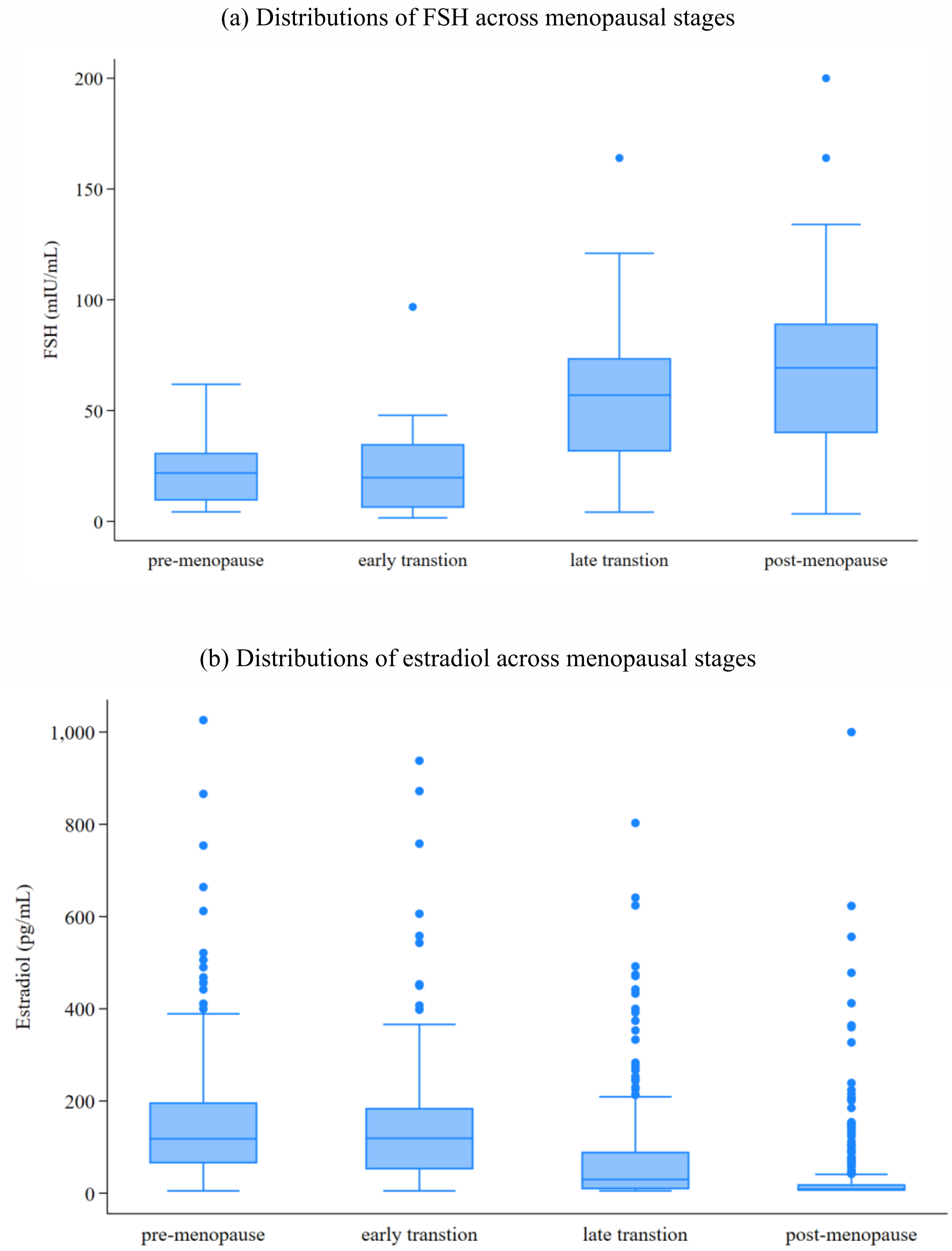

Figure S2. Predicted Probability of Sarcopenic Obesity by Menopausal Transition Stages at Fixed Ages (45 and 50 Years).

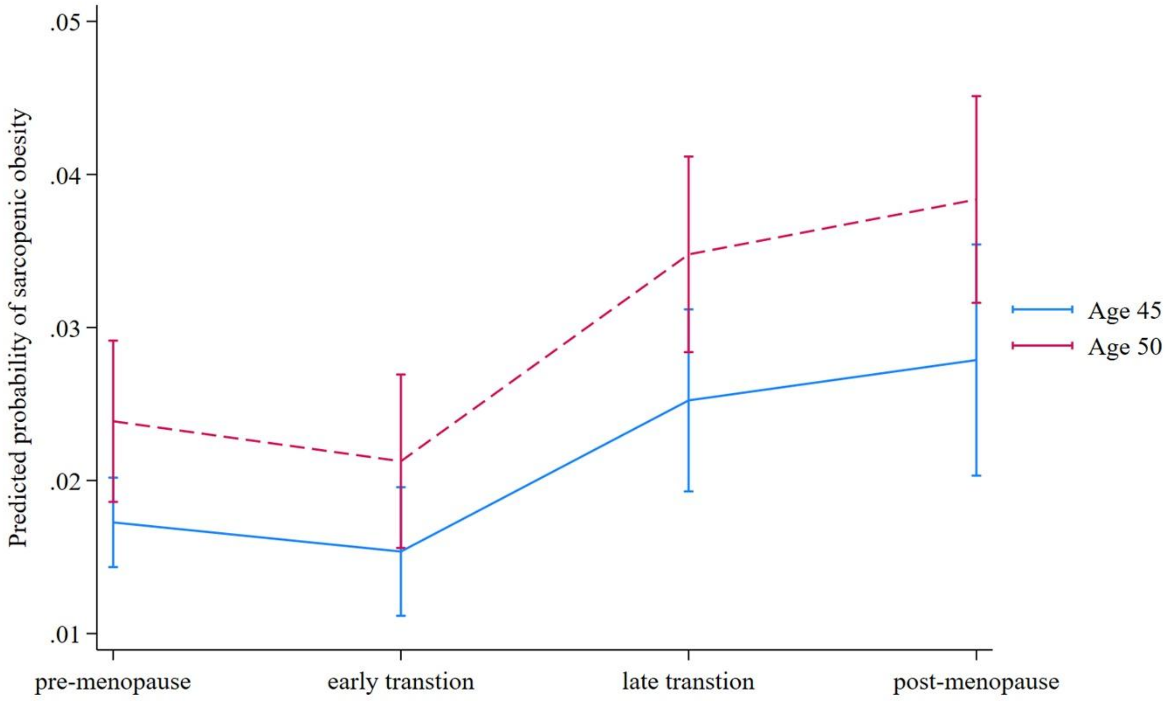

Figure S3. Predicted percentage body fat by years relative to the final menstrual period, estimated using cubic restricted spline models.

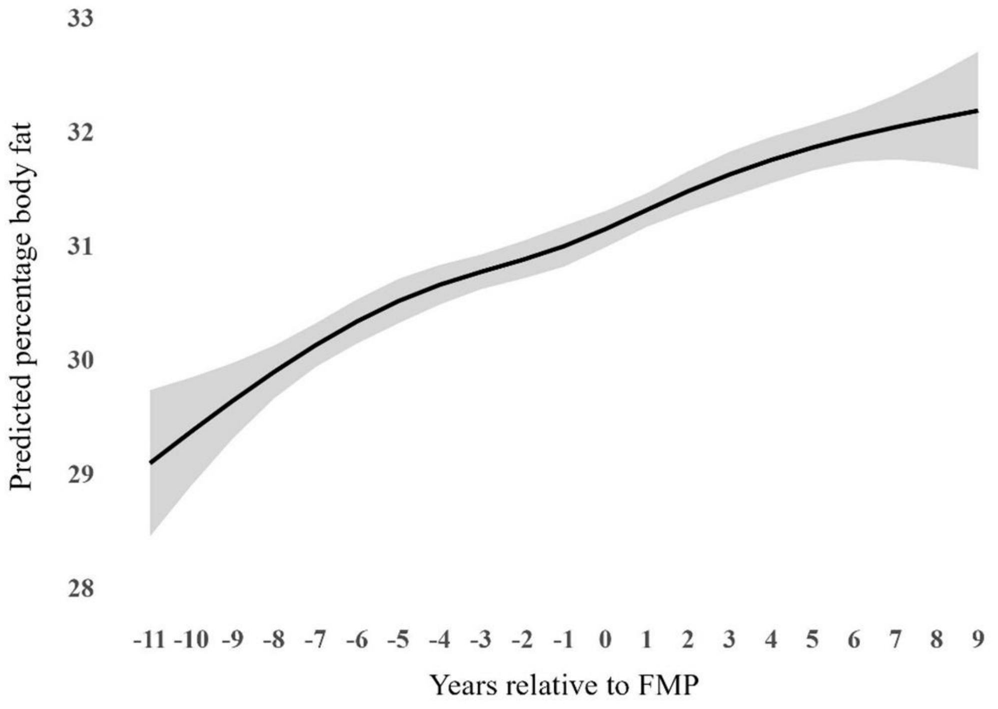

Figure S4. Predicted waist circumference relative to the final menstrual period, estimated using cubic restricted spline models.

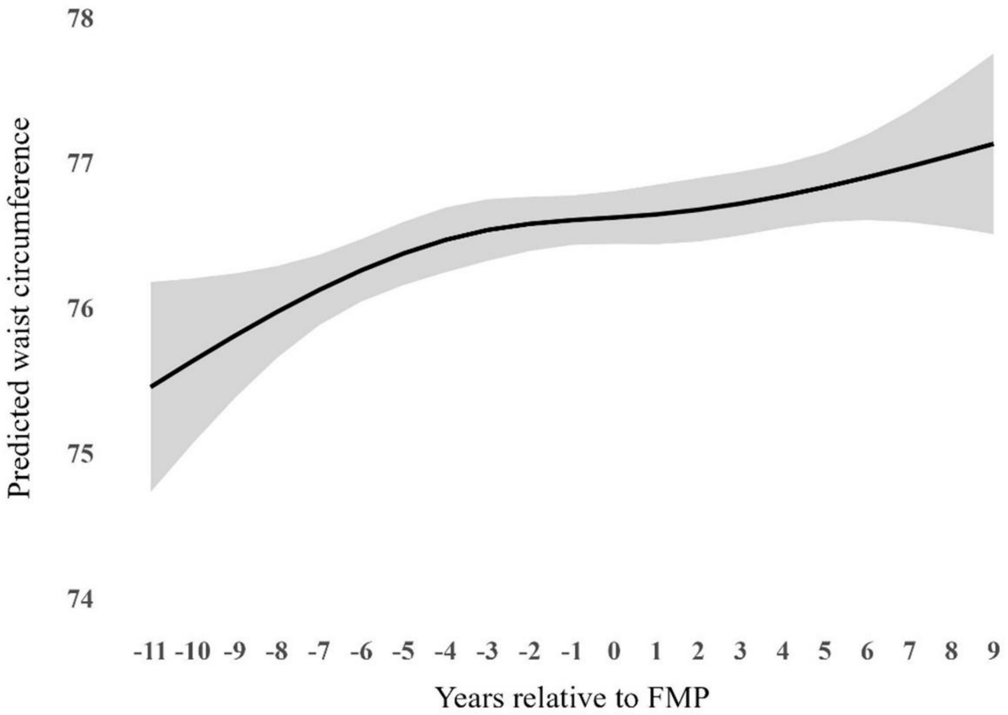

Supplement: Supplementary file 1 [file nutrients-17-03238-s001.zip › nutrients-3888618-supplementary.pdf]
